# Effects of Vitamin D Supplementation on Adipose Tissue Inflammation and NF-κB/AMPK Activation in Obese Mice Fed a High-Fat Diet

**DOI:** 10.3390/ijms231810915

**Published:** 2022-09-18

**Authors:** Eugene Chang

**Affiliations:** Department of Food and Nutrition, Gangneung-Wonju National University, Gangneung-si 25457, Gangwon-do, Korea; echang@gwnu.ac.kr; Tel.: +82-33-640-2338

**Keywords:** adenosine monophosphate-activated protein kinase (AMPK), adipose tissue, inflammation, nuclear factor-kappa B (NF-κB), NOD-, LRR- and pyrin domain-containing protein 3 (NLRP3), obesity, vitamin D

## Abstract

Adipose tissue expansion is strongly associated with increased adipose macrophage infiltration and adipocyte-derived pro-inflammatory cytokines, contributing to obesity-associated low-grade inflammation. Individuals with vitamin D deficiency have an increased prevalence of obesity and increased circulating inflammatory cytokines. However, the effect of vitamin D supplementation on obesity-induced inflammation remains controversial. Male C57BL/6J mice received a low-fat (10% fat) or high-fat (HF, 60% fat diet) containing 1000 IU vitamin D/kg diet, or HF supplemented with 10,000 IU vitamin D/kg diet for 16 weeks (*n* = 9/group). Vitamin D supplementation did not decrease HF-increased body weight but attenuated obesity-induced adipose hypertrophy and macrophage recruitment as demonstrated by the number of crown-like structures. Vitamin D supplementation significantly reduced the mRNA expression of CD11c, CD68, and iNOS, specific for inflammatory M1-like macrophages, and decreased serum levels of NO. In addition, significant reductions in pro-inflammatory gene expression of IL-6, MCP-1, and TNFα and mRNA levels of ASC-1, CASP1, and IL-1β involved in NLRP3 inflammasome were found in obese mice supplemented with vitamin D. Vitamin D supplementation significantly increased obesity-decreased AMPK activity and suppressed HF-increased NF-κB phosphorylation in adipose tissue from obese mice. These observed beneficial effects of vitamin D supplementation on adipose tissue expansion, macrophage recruitment, and inflammation might be related to AMPK/NF-κB signaling.

## 1. Introduction

Obesity is the consequence of a chronic imbalance between energy intake and energy expenditure, which is characteristic of excessive fat accumulation in adipose tissue [1]. Adipocyte expansion stimulates macrophage infiltration in adipose tissue, exhibiting a crown-like structure (CLS) surrounding dead adipocytes [2,3]. Macrophage infiltration into expanded adipose tissue leads to macrophage polarization from anti-inflammatory macrophage M2 to pro-inflammatory macrophage M1 and the production and release of adipocyte-derived biologically active proteins, such as interleukin-6 (IL-6), monocyte chemoattractant protein-1 (MCP-1), and tumor necrosis factor α (TNFα) [4]. These alterations in adipose tissue size and function might link obesity and obesity-associated metabolic complications [5]. Thus, reducing adipose tissue size and dysfunction and its associated inflammation might be an effective strategy for preventing obesity and its related health outcomes.

A negative association between adenosine monophosphate-activated protein kinase (AMPK) activation and inflammation has been reported in adipose tissue and macrophages in humans with obesity [6]. In adipose tissue from diet-induced or genetically modified obese mice, AMPK activation significantly downregulates pro-inflammatory mRNA expression of IL-6, MCP-1, and TNFα [7,8]. Evidence shows that AMPK signaling inhibits the activation of nuclear factor-kappa B (NF-κB), a critical regulator of innate immunity and inflammation. Increased nuclear translocation of NF-κB, NF-κB activation, and transcription of inflammatory genes in adipose tissue macrophages (ATM) from obese animals have been reported [9,10]. In addition, AMPK signaling blocks NOD-, LRR- and pyrin domain-containing protein 3 (NLRP3) inflammasome activation [11]. The NLRP3 inflammasome is a multiprotein complex consisting of NLRP3, apoptosis-associated speck-like protein containing a caspase activation recruitment domain (ASC), and procaspase-1 (CASP-1) and triggers the activation of CASP-1 and the secretion of pro-inflammatory interleukin-1β (IL-1β) and interleukin-18 (IL-18) [12,13]. Accumulating evidence indicates that adipose tissue of obese subjects has higher NLRP3 compartments and IL-1β expression compared to that of lean individuals, showing positive correlation between NLRP3 signaling in adipose tissue and obesity [14,15]. Furthermore, NF-κB activation mediates NLRP3 inflammasome and systematic inflammation by orchestrating the release of inflammatory cytokines [16]. Understanding the underlying molecular mechanism of how the AMPK/NF-κB pathway and related NLRP3 inflammasome affect obesity and obesity-induced inflammation might lead to preventive strategy for obesity-mediated metabolic diseases.

Numerous studies demonstrate that obesity is associated with lower circulating 25-hydroxyvitamin D ([25(OH)D]) levels [17,18,19]. However, the effects of vitamin D supplementation on obesity has thus far been inconclusive. A 12-week intervention study illustrates that elevated 25(OH)D levels after supplementation are negatively associated with body mass index in people over 64 [20]. Several studies, including clinal studies, systemic review, and meta-analyses have not shown the beneficial effects of vitamin D supplementation on weight reduction or adiposity [21,22,23,24]. Regarding the effect of vitamin D on obesity-associated inflammation, increased serum concentrations of inflammatory cytokines, such as IL-6 and TNFα, have been reported in obese subjects with a vitamin D deficiency [25,26]. Surprisingly, a systematic review with meta-analysis illustrates that vitamin D supplementation does not decrease inflammatory biomarkers in the blood of obese and overweight subjects [27]. Contrary to the inconsistent data from the meta-analysis, treatment with the biologically active form of vitamin D, 1,25-dihydroxyvitamin D ([1,25(OH)2D]), calcitriol), suppresses NF-κB pathway-induced inflammatory markers in human adipocytes and 3T3-L1 adipocytes [28,29,30,31]. Unlike the detrimental effects of vitamin D deficiency on obesity and its associated inflammation, data on the influence of vitamin D supplementation remain conflicting. In addition, the molecular mechanism by which dietary vitamin D supplementation affects NF-κB signaling in hypertrophic and inflamed adipose tissue from obese animals has not yet been fully delineated.

The specific aim of this current study was to investigate the effects of vitamin D supplementation on obesity and obesity-associated inflammation in mice fed a high-fat diet (HF). To demonstrate the molecular mechanism by which vitamin D supplementation influences adipose tissue formation and function, the activation of AMPK and NF-κB was determined in adipose tissue from obese mice.

## 2. Results

### 2.1. Effects of Dietary Vitamin D Supplementation on Body Weight and Food Intake in HF-Fed Obese Mice

**Figure 1:**
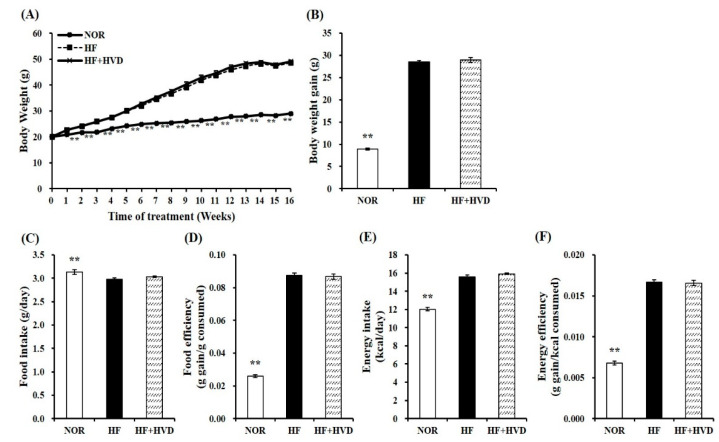
Effect of vitamin D supplementation on body weight and food intake. (**A**) Changes of body weight (g) during 16-week diet supplementation, (**B**) body weight gain (g), (**C**) food intake (g consumed/day), (**D**) food efficiency (g gain/g consumed) = body weight gain (g)/total food consumption (g), (**E**) energy intake (kcal/day), and (**F**) energy efficiency (g gain/kcal consumed) = body weight gain (g)/total energy intake (kcal). Values are expressed as the mean ± SEM (*n* = 9/group). NOR, 10% fat diet with 1000 IU vitamin D; HF, 60% fat diet with 1000 IU vitamin D; HF + HVD, 60% fat diet with 10,000 IU vitamin D. ** *p* < 0.01 compared to the HF group.

After 1 week of acclimation, 6-week-old male C57BL/6J mice were fed a 10% fat containing 1000 IU vitamin D/kg diet (NOR), or a 60% fat diet containing 1000 IU vitamin D/kg diet (HF), or HF supplemented with 10,000 IU vitamin D/kg diet (HF + HVD) for 16 weeks (*n* = 9/group). There was no significant difference between the three groups at the start of the experiment (week 0; Figure 1A). The statistical difference in body weight (BW) between the NOR group and HF group started from week 2 of the experiment (*p* < 0.01). As shown in Figure 1A,B, the final BW and BW gain in HF-fed animals were statistically higher than those in NOR-fed mice, indicating that chronic HF consumption led to obesity, as expected (*p* < 0.01). Vitamin D supplementation in HF did not prevent obesity as demonstrated by no statistical differences in weekly BW change (Figure 1A), final BW (Figure 1A), and BW gain (Figure 1B) between HF and HF + HVD groups. In addition, no statistical differences in food intake (Figure 1C), food efficiency (Figure 1D), energy intake (Figure 1E), and energy efficiency (Figure 1F) were observed in vitamin D-supplemented obese mice (HF + HVD) compared to HF-fed obese mice.

### 2.2. Effects of Vitamin D Supplementation on Serum Metabolic Parameters and Serum 25-Hydroxy Vitamin D (25(OH)D) Concentration

**Figure 2:**
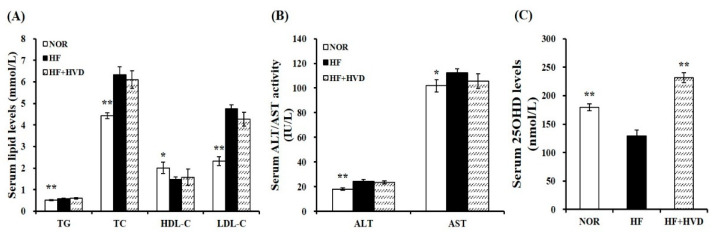
Effect of vitamin D supplementation on serum metabolic parameters. (**A**) Serum levels of TG, TC, HDL-C, and LDL-C. LDL-C = TC − HDL-C − (TG/5). (**B**) Serum ALT and AST activities. (**C**) Serum 25OHD amounts. Values are expressed as the mean ± SEM (*n* = 9/group). NOR, 10% fat diet with 1000 IU vitamin D; HF, 60% fat diet with 1000 IU vitamin D; HF + HVD, 60% fat diet with 10,000 IU vitamin D. ALT, alanine transaminase; AST, aspartate aminotransferase; HDL-C, high-density lipoprotein-cholesterol; LDL-C, low-density lipoprotein-cholesterol; TC, total cholesterol; TG, triglyceride. * *p* < 0.05; ** *p* < 0.01 compared to the HF group.

To investigate the association between diet composition and vitamin D status, serum 25(OH)D concentration, an indicator of vitamin D status, was measured using a commercial enzyme-linked immunosorbent assay (ELISA) kit. As shown in Figure 2C, NOR induced a significant increase in serum 25OHD levels, compared to HF. In HF-fed animals, vitamin D supplementation significantly increased the serum 25(OH)D concentration (*p* < 0.01).

Next, the serum activities of alanine transaminase (ALT) and aspartate aminotransferase (AST) levels were analyzed to determine whether dietary vitamin D level (10,000 IU/kg HF) affected liver toxicity. At the end of the 16-week experimental period, the serum ALT and AST activities were not statistically different between HF and HF + HVD. Therefore, in this study, vitamin D supplementation in HF did not induce any harmful damage to the liver (Figure 2B).

The influence of vitamin D supplementation on obesity-related dyslipidemia was investigated by measuring the levels of serum triglyceride (TG), total cholesterol (TC), low-density lipoprotein-cholesterol (LDL-C), and high-density lipoprotein-cholesterol (HDL-C). HF significantly increased the serum concentrations of TG, TC, and LDL-C by 13.9%, 43.1%, and 104.5%, respectively and significantly reduced the circulating HDL-C contents by 26.3%, compared to the NOR group (*p* < 0.05). However, there was no remarkable change in the serum lipid profile between HF and HF + HVD (Figure 2A).

### 2.3. Effects of Vitamin D Supplementation on Adipocyte Enlargement and Adipogenic Gene Expression in Epididymal White Adipose Tissue (eWAT) from HF-Induced Obese Mice

**Figure 3:**
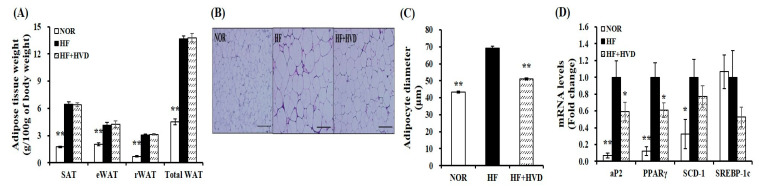
Effect of vitamin D supplementation on adipose tissue weight and adipocyte size. (**A**) White adipose tissue (WAT) weights. (**B**) Representative hematoxylin and eosin (H&E)-stained epidydimal sections (scale bars, 200 μm; magnification, 400×). (**C**) Average adipocyte diameter of epididymal WAT (eWAT). (**D**) Gene expression of adipogenesis in eWAT. Gene expression of β-actin, as a reference gene was used for normalization, and data were presented as fold change compared to the HF group. Values are expressed as the mean ± SEM (*n* = 9/group). NOR, 10% fat diet with 1000 IU vitamin D; HF, 60% fat diet with 1000 IU vitamin D; HF + HVD, 60% fat diet with 10,000 IU vitamin D. * *p* < 0.05; ** *p* < 0.01 compared to the HF group.

At the end of the 16-week experimental period, the weights of subcutaneous adipose tissue (SAT), eWAT, retroperitoneal white adipose tissue (rWAT), and total white adipose tissue (WAT) from obese mice fed HF were significantly increased compared to NOR-fed animals (*p* < 0.01; Figure 3A). Next, adipocyte diameter from eWAT of lean and obese mice was measured and presented as the fold difference compared to the HF group (Figure 3B,C). Adipocyte diameters in NOR-fed mice were statistically smaller than that of HF-fed obese mice (Figure 3B). HF-increased adipocyte size was significantly reduced by 26.3% in the HF + HVD group (*p* < 0.01; Figure 3C).

Regarding the inhibitory effect of vitamin D supplementation on adipocyte hypertrophy, gene expression involved in adipogenesis in eWAT was measured by real-time quantitative polymerase chain reaction (RT-qPCR). As shown in Figure 3D, adipocyte protein 2 (aP2), peroxisome proliferator-activated receptor γ (PPARγ), and stearoyl-CoA desaturase-1 (SCD-1) mRNA levels were significantly higher in eWAT from HF-fed obese mice compared to NOR-fed animals, whereas the supplementation of HF with vitamin D showed a significant reduction in aP2 and PPARγ gene expression by 41% and 39%, respectively, compared to HF (*p* < 0.05). Furthermore, 23% and 47% reduction in SCD-1 and sterol regulatory element binding protein-1c (SREBP-1c) mRNA levels were observed in eWAT from mice fed a HF diet supplemented with vitamin D without a statistical difference (Figure 3D).

### 2.4. Effects of Vitamin D Supplementation on Adipose Tissue and Systematic Inflammation

**Figure 4:**
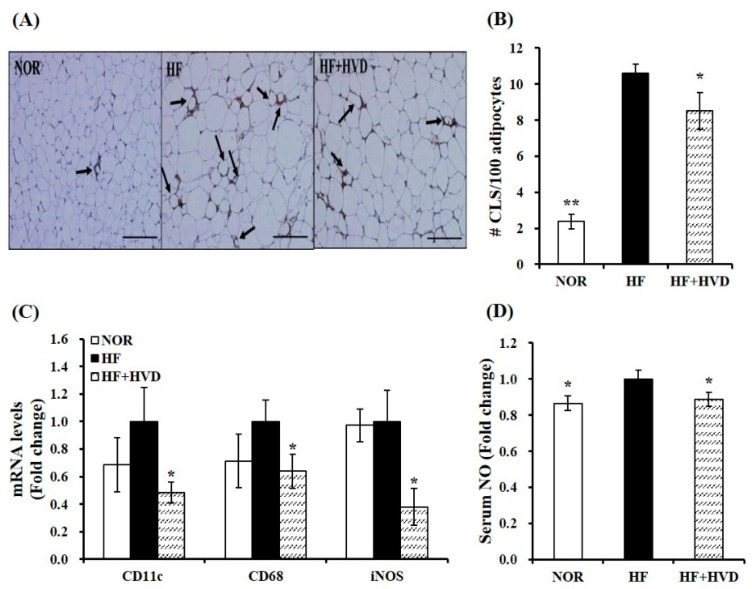
Influence of vitamin D supplementation on adipose tissue macrophage accumulation and polarization in epididymal white adipose tissue (eWAT) of obese mice. (**A**) Representative images of F4/80-immunostained eWAT (scale bars, 200 μm; magnification, 400×). The black arrows represent a crown-like structure (CLS). (**B**) The mean CLS number was observed under a microscope, quantified from multiple histologic sections, and expressed as CLS number per 100 adipocytes. (**C**) mRNA levels related to M1/M2 macrophage polarization were determined by RT-qPCR, normalized to that of β-actin, and expressed as the fold change compared to the HF group. (**D**) Serum nitric oxide (NO) amount was analyzed using a Griess reagent kit and expressed as the fold difference relative to the HF group. Values are expressed as the mean ± SEM (*n* = 9/group). NOR, 10% fat diet with 1000 IU vitamin D; HF, 60% fat diet with 1000 IU vitamin D; HF + HVD, 60% fat diet with 10,000 IU vitamin D. * *p* < 0.05; ** *p* < 0.01 compared to the HF group.

To identify whether vitamin D supplementation might have beneficial effects on local and systematic inflammation in HF-fed obese mice, macrophage infiltration in eWAT was detected by F4/80 immunohistochemical staining and quantified by counting CLS density as CLS number/100 adipocytes. An indicator of the pro-inflammatory process, CLS is macrophages that originated from blood monocytes and surrounding dead adipocytes [2]. As shown in Figure 4A, HF-fed obese mice had significantly more F4/80-positive adipocytes, as demonstrated by macrophages surrounding adipocytes (CLS). The mean number of CLS per 100 adipocytes in eWAT of HF-fed obese mice was significantly higher than that of the NOR group. This result shows that obese animals had more dead adipocytes and macrophage infiltration in eWAT depots. In addition, vitamin D supplementation significantly reduced HF-increased CLS number by 19.9% (*p* < 0.05; Figure 4B). It suggests that dietary vitamin D supplementation might alleviate macrophage recruitment into adipose tissue.

ATM accumulation is closely associated with a phenotypic switch to M1 polarization [32]. Next, mRNA levels related to ATM polarization in eWAT were measured by RT-qPCR. Vitamin D supplementation to HF significantly suppressed gene expression of M1 macrophage markers CD11c (integrin alpha X), cluster of differentiation 68 (CD68), and inducible nitric oxide synthase (iNOS) by 52%, 36%, and 62%, respectively (*p* < 0.05; Figure 4C). Circulating nitric oxide (NO) levels are closely associated with inflammation due to their production by iNOS [33]. As described in Figure 4D, vitamin D supplementation significantly suppressed HF-increased serum NO production by 11.1% (*p* < 0.05), indicating that dietary vitamin D supplementation might prevent HF-induced systematic inflammation.

### 2.5. Influence of Vitamin D Supplementation on Gene Expression Related to Procytokine and NLRP3 Inflammasome Components in eWAT and Serum Pro-Inflammatory Cytokine Level

**Figure 5:**
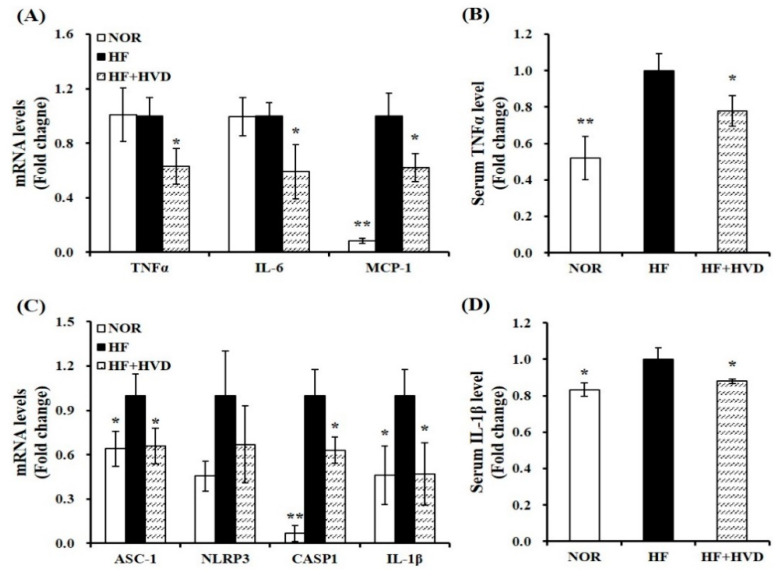
Effect of vitamin D supplementation on pro-inflammatory cytokines and NLRP3 inflammasome. mRNA levels related to (**A**) pro-inflammatory cytokines and (**C**) NLRP3 inflammasome components in epididymal white adipose tissue (eWAT) were measured by RT-qPCR, normalized to β-actin, and expressed as the fold change compared to the HF group. Serum levels of (**B**) TNFα and (**D**) IL-1β were quantified using commercial colorimetric ELISA kits, normalized to their respective protein concentrations, and presented as the fold change compared to the HF group. Values are expressed as the mean ± SEM (*n* = 9/group). NOR, 10% fat diet with 1000 IU vitamin D; HF, 60% fat diet with 1000 IU vitamin D; HF + HVD, 60% fat diet with 10,000 IU vitamin D. * *p* < 0.05; ** *p* < 0.01 compared to the HF group.

To evaluate the effect of the dietary vitamin D level on eWAT expansion-related local and systematic inflammation, the production and release of adipocyte-derived pro-inflammatory cytokines were measured in serum or eWAT using RT-qPCR and commercial ELISA kits. Vitamin D supplementation significantly decreased the gene expression of TNFα, IL-6, and MCP-1 by 37%, 41%, and 38%, respectively compared to the HF group (*p* < 0.05; Figure 5A). Consistent with the measured mRNA levels related to the production of adipocyte-derived pro-inflammatory peptides, HF supplemented with vitamin D significantly reduced serum TNFα concentration by 22%, compared to HF (*p* < 0.05; Figure 5B).

Next, the mRNA levels involved in NLRP3 components and serum IL-1β concentration were analyzed to determine whether vitamin D decreased NLRP3 inflammasome-associated pro-inflammatory cytokine production, ATM, and systematic inflammation. Long-term HF intake increased the mRNA levels of ASC-1, NLRP3, CASP1, and IL-1β, whereas they were downregulated by vitamin D supplementation by 34%, 33%, 37%, and 53% (Figure 5C). In addition, vitamin D supplementation significantly inhibited serum IL-1β levels by 12% (Figure 5D). These results suggest that vitamin D supplementation could counteract HF-increased gene expression of NLRP3 inflammasome components and systematic inflammation.

### 2.6. Effect of Vitamin D Supplementation on AMPK/NF-κB Activation

**Figure 6:**
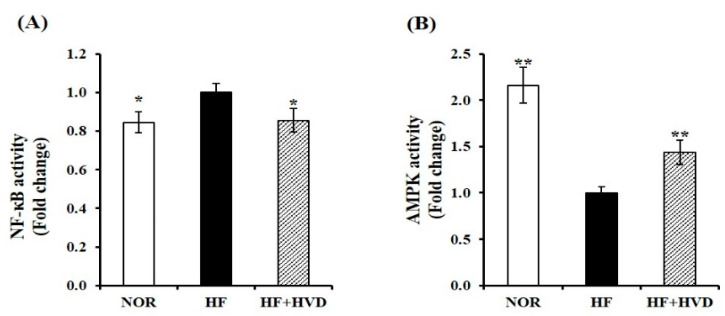
Influence of vitamin D supplementation on AMPK and NF-κB activities. Commercial ELISA kits were used to measure nuclear (**A**) NF-κB activity and (**B**) AMPK activation in epididymal WAT (eWAT) from obese mice. Results were normalized to their relative protein levels and presented as the fold difference compared to the HF group. Values are expressed as the mean ± SEM (*n* = 9/group). NOR, 10% fat diet with 1000 IU vitamin D; HF, 60% fat diet with 1000 IU vitamin D; HF + HVD, 60% fat diet with 10,000 IU vitamin D. * *p* < 0.05; ** *p* < 0.01 compared to the HF group.

Two key regulators of inflammation, AMPK and NF-κB, have been reported in association with NLRP3 inflammasome activation [34,35]. To investigate whether the anti-inflammatory effect of vitamin D supplementation is related to AMPK/NF-κB activation, the nuclear protein levels of phosphorylated 65-NF-κB (pS536) and total p65-NF-κB and AMPK kinase were analyzed in eWAT using commercial ELISA kits. As shown in Figure 6A, in eWAT nuclear extracts from HF-fed obese mice, the p65 subunits of NF-κB were significantly higher than those of lean mice (*p* < 0.05). HF-induced nuclear NF-κB activation was significantly inhibited by vitamin D supplementation by 14% (*p* < 0.05). By contrast, AMPK activity was significantly increased by 1.4-fold with vitamin D supplementation compared to HF feeding (*p* < 0.05; Figure 6B).

## 3. Discussion

A negative association between vitamin D status and adipose expansion and inflammation has been reported in obese participants and rodent animals [17,18,19,36]. Nevertheless, the beneficial effects of vitamin D supplementation on adiposity and pro-inflammatory cytokines are controversial. This study aimed to demonstrate the effects of dietary vitamin D supplementation on HF-induced weight gain, adipose hypertrophy, adipose macrophage polarization and recruitment, and inflammation in obese mice. In the current study, 16-week supplementation of vitamin D inclusion in HF (10,000 IU/kg) did not change the HF-increased BW but alleviated the HF-increased adipocyte size with reduced ATM. Serum levels of inflammatory markers, including IL-1β and TNFα were significantly downregulated by vitamin D supplementation, along with decreased mRNA levels involved in M1 macrophage polarization, pro-inflammatory cytokines, and NLRP3 components in eWAT. In addition, HF-increased AMPK activation and nuclear NF-κB phosphorylation in adipose tissue were significantly decreased by vitamin D supplementation.

An increase in adipose size (hypertrophy) and number (hyperplasia) occurs in response to excessive energy surplus [1]. In the present study, 16 weeks of HF feeding induced a significant increase in BW, BW gain, adipose tissue weights, and adipocyte diameter. In addition, HF-fed obese mice displayed lipid abnormalities such as higher serum levels of TG, TC, and LDL-C and a lower circulating HDL-C concentration. However, this obesity phenotype and obesity-associated dyslipidemia were not changed by 10,000 IU vitamin D supplementation in obese mice. These results corroborate previous clinical studies and a systematic and meta-analysis showing no observed favorable effect of vitamin D supplementation on adiposity and weight reduction in obese participants [21,22,24]. Consistent with these human studies, no beneficial effects of 10,000, 15,000, or 250,000 IU vitamin D supplementation on adiposity in obese mice have been reported [37,38,39]. In addition, a systematic review and meta-analysis of randomized clinical trials illustrates that vitamin D supplementation does not influence blood lipid profiles in adults with metabolic syndrome [40]. The current dose of 10,000 IU vitamin D/kg HF did not induce further liver toxicity, as demonstrated by no change in serum ALT and AST activities compared to HF. These data suggest that 16 weeks of vitamin D supplementation did not improve diet-induced obesity and lipid abnormality.

Adipose tissue is an active endocrine organ, and increased adipocyte size produces and secretes adipokines involved in metabolic regulation and ATM, as demonstrated by CLS formation [2,4]. Furthermore, a study demonstrates the molecular mechanisms by which hypertrophic adipocytes might be a crucial determinant of pro-inflammatory cytokines [41]. In the present study, vitamin D supplementation significantly suppressed HF-increased adipocyte diameter and significantly reduced the adipogenic mRNA levels of aP2 and PPARγ. The transcriptional factor PPARγ is a major regulator of adipocyte differentiation from which preadipocytes become mature adipocytes and accumulates lipids [42]. Lipid-binding protein aP2 has been shown to play a critical role in fat accumulation and inflammation in relation to NF-κB activity [43]. As with the result of adipocyte diameter, 16 weeks of vitamin D supplementation significantly suppressed HF-increased CLS number in eWAT. In expanded adipose tissue, macrophage recruitment that characterizes CLS is associated with a phenotype switch to pro-inflammatory M1 macrophage [2,32]. Next, the gene expression of M1 gene markers in eWAT was determined by RT-qPCR. Significant reductions in the mRNA levels of M1 macrophage markers such as CD11c, CD68, and iNOS were observed in eWAT of obese mice supplemented with vitamin D. In the obese state, M1 macrophages secrete pro-inflammatory adipokines including IL-6, TNFα, and MCP-1, and stimulates NO production through M1 macrophages-induced iNOS [4,32,33]. The HF + HVD group had a significantly lower mRNA expression of IL-6, TNFα, and MCP-1 in eWAT compared to the HF group. In addition, vitamin D supplementation significantly inhibited HF-increased serum levels of TNFα and NO. Similarly, cholecalciferol supplementation for 10 weeks significantly reduces IL-6, TNFα, and CD11c mRNA levels in eWAT from obese C57BL/6J mice fed an HF diet (45% fat) [28]. Another study in which an HF/high-sucrose diet was supplemented with 15,000 IU cholecalciferol for 15 weeks has shown a significant decrease in MCP-1 mRNA expression in inguinal adipose tissue [38]. Therefore, it suggests that dietary vitamin D supplementation for 16 weeks could alleviate obesity-induced ATM and inflammation by decreasing macrophage accumulation and M1 polarization in eWAT.

Adipose tissue remodeling and dysfunction, as described by low-grade inflammation and macrophage recruitment, is closely associated with the synthesis and release of pro-inflammatory cytokines [2,4]. These adipokines could promote systematic metabolic inflammation by the NLRP3 inflammasome [14,15]. The molecular mechanism by which NLRP3 regulates inflammation and macrophage activity involves the activation of CASP-1, which cleaves IL-1β [12,13]. A study investigating the role of NLRP3 inflammasome in obesity and obesity-induced inflammation shows that the mRNA levels of IL-1β and NLRP3 in the visceral adipose tissue are positively correlated with BW of C57BL/6 mice. In this study, F4/80-immunostained eWAT demonstrates that NLRP3 compartments are co-localized with ATM [44]. Genetic ablation of NLRP3 prevents HF-induced obesity, obesity-induced ATM, and inflammation [44,45,46]. Morbidly obese subjects with laparoscopic adjustable gastric banding have significantly lower SAT mRNA expression of IL-1β [47]. In the present study, 16 weeks of HF feeding significantly increased mRNA levels of ASC, CASP1, and IL-1β in eWAT and significantly raised the circulating IL-1β levels compared to NOR. NLRP3 mRNA expression in the HF group was 2.2-fold higher than in the NOR group without a statistical difference. Vitamin D supplementation significantly downregulated HF-induced gene expression of ASC, CASP1, and IL-1β involved in NLRP3 inflammasome compartments and decreased NLRP3 gene expression by 13% without a statistical difference. Serum IL-1β concentrations were alleviated by vitamin D supplementation in obese mice fed HF. Therefore, it is plausible to surmise that the reduction in obesity-induced ATM and systematic inflammation in the HF + HVD group might be associated with the inhibitory effect of vitamin D on NLRP3-related IL-1β levels.

Accumulating evidence indicates that obese individuals and HF-fed animals have enhanced activation of NF-κB, a central regulator of inflammation [9,10,48]. NF-κB, a transcription factor for pro-inflammatory cytokines, is normally inactive in the cytoplasm because of its interaction and complexation with inhibitor of κBa (IκBa). In response to inflammatory stimuli, IκB kinase (IKK) phosphorylates IκBα, promotes ubiquitin-dependent IκBα degradation, and releases NF-κB, which contributes to the nuclear translocation of NF-κB and the transcription of target genes, such as TNFα and IL-6 [49]. Inflammatory responsive NF-κB activation modulates obesity-associated macrophage recruitment in adipose tissue from diet-induced and genetically obese mice [10]. In addition to regulating various pro-inflammatory gene transcripts and ATM, NF-κB also plays an important role in obesity-induced inflammasome activation. NF-κB activity mediates the NLRP3 inflammasome and inflammation by upregulating NLRP3 and pro-IL-1β transcription and releasing these inflammatory cytokines [50]. In the present study, prolonged HF consumption induced significant induction of NF-κB activity in eWAT compared to NOR. Sixteen weeks of vitamin D supplementation significantly inhibited HF-increased NF-κB phosphorylation in eWAT from HF-fed obese mice, accompanied by alterations in CLS number, mRNA levels of pro-inflammatory cytokines, such as TNFα and IL-6, NLRP3-related compartments, ASC-1, CASP1, and IL-1β, and circulating IL-1β and TNFα levels. The molecular mechanisms by which vitamin D alleviates the obesity-associated inflammatory condition have been investigated using 1,25(OH)2D-treated human adipocytes and 3T3-L1 treated adipocytes. Consistent with the present study, 1,25(OH)2D treatment significantly suppresses macrophage-induced NF-κB activation in human adipocytes by increasing IκBα expression and reducing NF-κB p65 phosphorylation. In addition, 1,25(OH)2D-treated human adipocytes show significantly lower mRNA levels of MCP-1, IL-1β, and IL-6, and the release of chemokines/cytokines, including MCP-1 and IL-6 [31]. Similarly, 1,25(OH)2D-treated human preadipocytes inhibit NF-κB activation by increasing the IκBα protein levels, leading to 1,25(OH)2D-reduced secretion of MCP-1, IL-6, and IL-8 [30]. In a study by Marcotorchino et al., 1,25(OH)2D-induced NF-κB inactivation is involved in a decrease in inflammatory markers, such as IL-6, MCP-1, and IL-1β, in human adipocytes and 3T3-L1 adipocytes [29]. Therefore, it is postulated that the protective effects of vitamin D on adipose tissue inflammation and circulating inflammatory cytokines might be associated with the involvement of NF-κB-related inflammation.

Recent evidence has demonstrated that AMPK acts as an important intracellular energy metabolic switch regulating cellular energy homeostasis, adenosine triphosphate (ATP) generation, and energy-consuming anabolic pathways via glucose and fatty acid uptake, mitochondrial biogenesis, mitochondrial oxidation signaling, and glycolysis and fatty acid oxidation [51]. AMPK activity has emerged as a possible promising target for the prevention and treatment of obesity due to its role in adipogenesis, fatty acid oxidation, thermogenesis, and browning WAT [52]. With respect to regulating obesity-associated inflammation, inflamed adipose tissue from human participants with obesity has low AMPK activation [6]. In addition, AMPK activation antagonized body fat accumulation and inflammatory gene expression of IL-6, MCP-1, and TNFα in WAT from genetically obese and diet-induced obese animals [7,8]. The association between AMPK and NF-κB signaling has been investigated in a study using 3T3-L1 adipocytes [53]. AMPK activation alleviates cytokine-stimulated pro-inflammatory expression and secretion by inhibiting NF-κB p65 translocation from the cytosol to the nucleus and IKK and IκB phosphorylation [53]. Furthermore, the inhibitory effects of AMPK on NLRP3 expression and the secretion of inflammatory mediators have been reported. Notably, AMPK activation protects inflammation and adipose dysfunction and attenuates NLRP3 inflammasome activation [11,54]. In the current in vivo experimental animal study, 16 weeks of HF feeding significantly decreased AMPK phosphorylation compared to NOR. Vitamin D supplementation significantly increased HF-decreased AMPK phosphorylation in eWAT from obese animals, similar to the inhibitory effect of a vitamin D-insufficient HF diet on AMPK activity [36]. Given the close association between AMPK/NF-κB activities and inflammation, vitamin D supplementation-reduced ATM infiltration and systematic inflammation might be associated with the AMPK/NF-κB pathway in eWAT from HF-fed obese mice.

The limitations of this present study were the gender bias and the small sample size. Only male mice (*n* = 9/group) were used in the current study to eliminate confounding factors such as reproductive cycles and hormone fluctuations. Application to female animals with a large sample number is warranted to investigate the role of dietary vitamin D supplementation on obesity-related adipose tissue inflammation in women. In addition, the direct effects of vitamin D supplementation on adipose tissue were not determined in this study, nor were the effects of vitamin D supplementation in NOR (NOR + HVD) used; thus, whether there was an improvement in vitamin D metabolism in adipose tissue by vitamin D supplementation cannot be stated.

In conclusion, the present study demonstrated that vitamin D supplementation did not improve HF-induced BW gain but ameliorated HF-increased adipose size, macrophage recruitment in eWAT, and mRNA transcripts and secretion of pro-inflammatory cytokines in obese mice without changing food intake. Vitamin D supplementation significantly increased the mRNA expression of ASC-1, CASP1, and IL-1β, which comprise the NLRP3 inflammasome, and significantly increased the circulating IL-1β levels and AMPK/NF-κB activities in eWAT. Therefore, it is postulated that dietary vitamin D supplementation might be a good strategy for alleviating obesity-induced adipose tissue inflammation and systematic inflammation.

## 4. Materials and Methods

### 4.1. Animals and Diets

All animal experiments were conducted in accordance with the National Institutes of Health (NIH) international guidelines and approved by the Institutional Animal Care and Use Committee (IACUC) of Ewha Womans University (IACUC No. 20-039). Male C57BL/6J mice (5-week-old, *n* = 27) were obtained from SaeronBio, Inc. (Uiwang-si, Gyeonggi-do, Korea) and housed in an ultraviolet-B-light-free environment on a 12/12-h light/dark cycle at 22 ± 1 °C and 55 ± 5% ambient humidity. After 1-week acclimatization with ad libitum water and a normal diet (D12450, Research Diets, Inc., New Brunswick, NJ, USA), a total of 27 mice were randomly divided into three experimental groups (*n*= 9/group) as follows: (1) a normal diet (NOR, 10% fat diet with 1000 IU vitamin D/kg diet), (2) a 60% high-fat diet (HF, 60% fat diet with 1000 IU vitamin D/kg diet, D12492), or (3) HF supplemented with 10,000 IU vitamin D/kg diet (HF + HVD). All diets were provided by Research Diets, Inc. Based on previous studies, dietary vitamin D dosages of adequacy or supplementation without harmless effects were chosen [36,39,55,56]. During the 16-week experimental period, BW and feed intake were monitored weekly. Following 16 weeks of dietary supplementation, mice were fasted overnight and euthanized by CO_2_ inhalation. Blood was collected by cardiac puncture and spun at 2000× *g*, 4 °C for 20 min. Serum was stored at −70 °C. Adipose tissues were collected, snap frozen in liquid nitrogen, and stored at −70 °C until further analysis. 

### 4.2. Serum Lipid Profile Analyses

Serum ALT and AST activities were analyzed by enzymatic colorimetric assays (Sigma-Aldrich, St. Louis, MO, USA). Serum TC and TG concentrations were determined by commercial assay kits (Abcam, Cambridge, UK). The serum level of HDL-C was quantified using commercial kits (Asan Pharmaceutical Co., Ltd., Seoul, Korea). Serum LDL-C level was calculated using the formula of Friedewald [57]; LDL-C = TC − HDL-C − (TG/5).

### 4.3. Serum 25-Hydroxy Vitamin D (25(OH)D) Measurement

A 25(OH)D ELISA kit (Eagle Biosciences, Inc., Amherst, NH, USA) was employed to measure serum vitamin D status according to the manufacturer’s instructions. Briefly, serum samples were added to wells coated with specific anti-25(OH)-vitamin D2 and D3 antibody, followed by biotinylated vitamin D analogue and horseradish peroxidase (HRP)-conjugated streptavidin. During the incubation, the immunocomplex of vitamin D, biotin D, and HRP-conjugated streptavidin was formed. The reaction was terminated using an acidic ELISA stop solution. The absorbance was detected at 450 nm in a spectrophotometric microplate reader (Varioskan Flash, Thermo Scientific, Waltham, MA, USA).

### 4.4. Serum Nitric Oxide (NO) Measurement

A Griess reagent kit for nitrate determination (Thermo Scientific) was employed to measure serum NO production. In a microplate, serum samples were mixed with the Greiss reagent containing equal volumes of *N*-(1-naphthyl)ethylenediamine and sulfanilic acid. The absorbance of the nitrite-containing samples was measured at 548 nm using a spectrophotometric microplate reader (Thermo Scientific). The nitrate concentration was analyzed relative to the nitrite standard solution and presented as the fold change compared to the HF group.

### 4.5. Serum Tumor Necrosis Factor α (TNFα) Determination

The BioLegend LEGEND MAX™ mouse TNFα ELISA kit (BioLegend, Inc., San Diego, CA, USA) was used to measure serum TNFα concentration according to the manufacturer’s instructions. A pre-coated ELISA plate with a hamster monoclonal anti-mouse TNFα antibody was detected with a biotinylated rabbit polyclonal anti-mouse TNFα antibody. The absorbance was read at 450 nm using a microplate reader (Thermo Scientific). Serum TNFα was expressed as the fold difference relative to the HF group.

### 4.6. Serum Interleukin-1β (IL-1β) Analysis

Serum IL-1β concentration was determined using a mouse IL-1β ELISA kit (Abcam) based on the manufacturer’s instructions. This kit utilizes an IL-1β capture antibody recognized by a mouse IL-1β antibody-precoated 96-well plate, which forms an antibody-analyte sandwich complex. The application of tetramethylbenzidine (TMB) solution develops a blue color, and the color intensity is proportional to the serum IL-1β levels. After the addition of the stop solution, the color intensity was detected at 450 nm using a microplate reader (Thermo Scientific).

### 4.7. Histological Analysis

eWAT was fixed overnight in 10% neutral-buffered formalin (Sigma) at room temperature, embedded in paraffin, sectioned to 5-μm thickness using a microtome (Leica Microsystems, Wetzlar, Germany), and mounted on a glass microscope slide. For histological analysis, hematoxylin and eosin (H&E) staining was executed according to the standard histological procedure. Digital images of H&E-stained eWAT were taken with an Olympus IX51 inverted microscope (Tokyo, Japan) at 400× magnification. Adipocyte diameter was measured from a cross-sectional area of adipocytes from digital images captured from H&E-stained eWAT using ImageJ software (NIH, Bethesda, MD, USA).

### 4.8. Immunohistochemistry and Crown-Like Structure (CLS) Measurement

Histological sections of eWAT were deparaffinized in xylene, rehydrated in an ethanol serial, and incubated with anti-F4/80 antibody solution (epidermal growth factor (EGF)-like molecule containing mucin-like hormone receptor 1; SC-52664, Santa Cruz Biotechnology, Santa Cruz, CA, USA). A Polink-2 Plus HRP anti-rat 3, 3′-diaminobenzidine tetrahydro-chloride (DAB) detection kit (Golden Bridge International, Inc., Irvine, CA, USA), and Mayer hematoxylin (ScyTek, Logan, UT, USA) were utilized for CLS analysis, as previously described [36]. After acquiring digital images of stained eWAT sections (magnification, 400×; Olympus), adipose tissue inflammation was determined by counting the number of CLS defined as the circular region surrounded by adipocytes from multiple histologic sections. Results were expressed as the mean number of CLS per 100 adipocytes (mean ± SEM).

### 4.9. Real-Time Quantitative Polymerase Chain Reaction (RT-qPCR)

Ten milligrams of eWAT was homogenized in 1 mL of TRIzol (Invitrogen, Carlsbad, CA, USA). Total RNA was extracted using the RNeasy Lipid Tissue Mini Kit according to the manufacturer’s instructions (Qiagen, Hilden, Germany) and quantified by the NanoDrop1000 spectrophotometer (Thermo Scientific). The Moloney Murine Leukemia Virus (MMLV) Reverse Transcriptase Kit (Bioneer, Daejeon, Korea) was used to generate cDNA from 1 μg of RNA. RT-qPCR was performed using AccuPower 2X Greenstar qPCR Master Mix (Bioneer) on a Rotor-Gene Q thermocycler (Qiagen). Relative mRNA expression was normalized to that of β-actin as an internal control, calculated by the 2^−∆∆Ct^ method [58], and was expressed as the fold difference related to the HF group. Primers sequences used in RT-qPCR are presented in Table S1.

### 4.10. Nuclear Factor-Kappa B (NF-κB) Measurement

A nuclear extraction kit (Abcam) was utilized to extract nuclear proteins from eWAT according to the manufacturer’s instructions. A semi-quantitative measurement was employed to measure phospho-65-NF-κB (pS536) and total p65-NF-κB protein levels in nuclear proteins of eWAT using an NF-κB p65 (pS536 + Total) ELISA kit (Abcam). eWAT nuclear extracts were added to pre-coated microplate wells, followed by an antibody cocktail consisting of p65-NF-κB (pS536) and total p65-NF-κB. The blue color was proportional to eWAT p65-NF-κB protein concentration and was generated by the addition of TMB substrate and HRP-related catalyzation. After stopping the reaction with the stop solution, the absorbance was measured at 450 nm (Thermo Scientific). Values were expressed as the fold change of the HF group.

### 4.11. AMPK Activity

A CycLex AMPK Kinase Assay Kit (MBL Life Science, Nagano, Japan) was used to analyze the AMPK activity in eWAT. According to the manufacturer’s recommendations, anti-phospho-mouse IRS-1 S789 monoclonal antibody, HRP-conjugated anti-mouse IgG antibody, substrate reagent, and stop solution were serially applied to a mouse IRS-1 S789-pre-coated plate. Color intensity was determined at 450 nm with a plate reader (Thermo Scientific), normalized to protein concentration (BCA Protein Assay Kit, Thermo Scientific), and presented the fold difference compared to the HF group.

### 4.12. Statistical Analysis

All data were expressed as the mean ± SEM. Statistical analyses were carried out using SPSS statistical software version 25.0 (SPSS, Inc., Chicago, IL, USA). Statistical significance was analyzed using a one-tailed Student’s *t*-test to compare the findings relative to the HF group was defined at *p* < 0.05.

## Data Availability

Not applicable.

## Supplementary Materials

The following supporting information can be downloaded at: https://www.mdpi.com/article/10.3390/ijms231810915/s1.
