# Supplementary material for: Effects of Vitamin D Supplementation on Adipose Tissue Inflammation and NF-κB/AMPK Activation in Obese Mice Fed a High-Fat Diet
Source: Int J Mol Sci. 2022 Sep 18;23(18):10915. doi: 10.3390/ijms231810915 (PMC9506068; doi:10.3390/ijms231810915)
Supplement: Supplementary file 1 [file ijms-23-10915-s001.zip › ijms-1902500-supplementary.pdf]

International Journal of *Molecular Sciences*

Table S1. Primers used for real-time quantitative polymerase chain reaction (RT-qPCR).

Gene	Accession number	Primer sequences (5′- 3′)
aP2	NM_024406.2	F: CGA CAG GAA GGT GAA GAG CA<br>R: ATT CCA CCA CCA GCT TGT CA
ASC-1	NM_023258.4	F: TTA ATC CCA GCA ACC AGG AG<br>R: CTT GAG TTA GGC CAG CCT TG
β-actin	NM_007393	F: GGA CCT GAC AGA CTA CCT CA<br>R: GTT GCC AAT AGT GAT GAC CT
CASP1	NM_009807.2	F: CAC AGC TCT GGA GAT GGT GA<br>R: CTT TCA AGC TTG GGC ACT TC
CD11c	NM_001363985.1	F: TTC ATC TCC ACG TCA AGC CC<br>R: AGG ACC TTG GTG GCA TCT TG
CD68	NM_001291058.1	F: AAA GGC CGT TAC TCT CCT GC<br>R: GGA GCT GGT GTG AAC TGT GA
IL-1β	NM_008361.4	F: GCC CAT CCT CTG TGA CTC AT<br>R: AGG CCA CAG GTA TTT TGT CG
IL-6	NM_031168.2	F: CCT TCC TAC CCC AAT TTC CA<br>R: TAA CGC ACT AGG TTT GCC GA
iNOS	NM_001313922.1	F: CCA CAG CAA TAT AGG CTC AT<br>R: GGA TTT CAG CCT CAT GGT AA
MCP-1	NM_011333.3	F: AGG TCC CTG TCA TGC TTC TG<br>R: TCT GGA CCC ATT CCT TCT TG
NLRP3	NM_145827.3	F: ATG CTG CTT CGA CAT CTC CT<br>R: AAC CAA TGC GAG ATC CTG AC
PPARγ	NM_001127330.1	F: TTG ATT TCT CCA GCA TTT CT<br>R: TGT TGT AAG GCT GGG TCT TT
SCD-1	NM_009127.4	F: ATG GAT ATC GCC CCT ACG AC<br>R: GAT GTG CCA GCG GTA CTC AC
SREBP-1c	NM_001358315.1	F: GCC TGC TTG GCT CTT CTC TT<br>R: AGG TCA GCT TGT TTG CGA TG
TNFα	NM_013693.2	F: AGC ACA GAA AGC ATG ATC CG<br>R: GCC ACA AGC AGG AAT GAG AA

ASC, apoptosis-associated speck-like protein containing a CARD; aP2, adipocyte protein 2; CASP1, caspase-1; CD, cluster of differentiation; IL, interleukin; iNOS, inducible nitric oxide synthase; MCP-1, monocyte chemoattractant protein-1; NLRP3, NOD-, LRR- and pyrin domain-containing protein 3; PPARγ, peroxisome proliferator-activated receptor γ; SCD-1, stearoyl-CoA desaturase-1; SREBP-1c, sterol regulatory element-binding protein-1c; TNFα, tumor necrosis factor α.
